# Malaria amongst children under five in sub-Saharan Africa: a scoping review of prevalence, risk factors and preventive interventions

**DOI:** 10.1186/s40001-023-01046-1

**Published:** 2023-02-17

**Authors:** Jacob Owusu Sarfo, Mustapha Amoadu, Peace Yaa Kordorwu, Abdul Karim Adams, Thomas Boateng Gyan, Abdul-Ganiyu Osman, Immanuel Asiedu, Edward Wilson Ansah

**Affiliations:** University of Cape Coast, Cape Coast, Ghana

**Keywords:** Malaria, Children under five, Risk factors, Sub-Saharan Africa, Scoping review

## Abstract

**Introduction:**

Africa has a higher burden of malaria-related cases and deaths globally. Children under five accounted for over two-thirds of all malaria deaths in sub-Saharan Africa (SSA). This scoping review aims to map evidence of the prevalence, contextual factors and health education interventions of malaria amongst children under 5 years (UN5) in SSA.

**Method:**

Four main databases (PubMed, Central, Dimensions and JSTOR) produced 27,841 records of literature. Additional searches in Google, Google Scholar and institutional repositories produced 37 records. Finally, 255 full-text records were further screened, and 100 records were used for this review.

**Results:**

Low or no formal education, poverty or low income and rural areas are risk factors for malaria amongst UN5. Evidence on age and malnutrition as risk factors for malaria in UN5 is inconsistent and inconclusive. Furthermore, the poor housing system in SSA and the unavailability of electricity in rural areas and unclean water make UN5 more susceptible to malaria. Health education and promotion interventions have significantly reduced the malaria burden on UN5 in SSA.

**Conclusion:**

Well-planned and resourced health education and promotion interventions that focus on prevention, testing and treatment of malaria could reduce malaria burden amongst UN5 in SSA.

**Supplementary Information:**

The online version contains supplementary material available at 10.1186/s40001-023-01046-1.

## Introduction

Malaria has been a serious global public health concern in the past decades, especially in Africa and other highly endemic regions [1, 2]. The World Health Organisation (WHO) recorded about 241 million cases and 627,000 deaths globally in 2020 [1]. Africa has a higher burden of malaria-related cases accounting for 95 percent of global malaria cases in 2019. In the same year, the African region recorded 96 percent of global malaria mortality. Despite the efforts directed towards protecting people against malaria infection, the region accounted for 93 percent of all malaria deaths globally in 2020 [2]. Besides, children under five (UN5) accounted for over two-thirds of all malaria deaths in sub-Saharan Africa (SSA) [1].

In the last two decades, many policies and interventions have been implemented to control malaria at the global level. These policies and interventions have accounted for a 47 percent reduction in mortality rates amongst UN5 between 2000 and 2019 [3]. Despite this remarkable achievement, a child UN5 dies of malaria every two minutes. This means that much more must be done to protect vulnerable children, especially UN5 [3].

In SSA, the burden of malaria amongst UN5 years varies across various countries. For example, Malaria contributes to more than 30% of UN5 deaths in Nigeria, and more than 10% in Tanzania [2]. Perhaps, during this period UN5 are most vulnerable as they have lost maternal immunity and they have not yet developed specific immunity to infection. Besides, children UN5 are at highest risk for malaria infection and its related complication. Also, despite malaria is traditionally more prevalent in rural areas amongst UN5 [4], evidence shows the opposite in SSA, where malaria is more common in urban areas [5–7]. According to a recently published scoping review, risk factors associated with malaria infection amongst UN5 in SSA are the use of bed net and education status. However, only 13 studies were included in the review and these studies analysed secondary national surveys with limited contextual factors [2].

Furthermore, regarding possible interventions to reduce number of infections, a promising strategy is based on implementing community-based prevention and control through heath education in SSA [3]. UNICEF believes that health education and promotion policies resulted in increased insecticide-treated nets (ITNs) use among children under five in SSA, from 30 percent in 2014 to 54 percent in 2020. However, variations exist in the uptake of malaria interventions in SSA. For instance, from 2014 to 2020, less than 25 percent of children in Angola and Zimbabwe slept under ITNs. In contrast, over 80 percent of Guinea−Bissau and Niger utilised ITNs [3]. Perhaps, disparities in health education and promotion interventions have accounted for the variations in the uptake of ITNs amongst children in SSA [8]. Hence, it is pertinent to understand the role of health education and promotion interventions in preventing malaria amongst UN5 in SSA.

Therefore, this scoping review aims to map the evidence of malaria prevalence and contextual factors amongst UN5 in SSA. In addition, this review aims to map evidence on health education and promotion targeting malaria amongst UN5. This review will help inform future studies, health education and promotion interventions targeting malaria in UN5 in SSA for improved health outcomes amongst this vulnerable population.

## Methods

This scoping review was conducted according to the guidelines outlined by Arksey and O’Malley [9]. The steps recommended by Arksey and O’Malley include: identifying and stating the research questions, identifying relevant studies, study selection, data collection, summary and synthesis of results and consultation. The research questions for this scoping review included: (1) what is the prevalence of malaria amongst children UN5 in SSA? (2) What are the risk factors associated with malaria infection amongst children UN5 in SSA? and (3) What are the health education and promotion interventions reported by studies to prevent malaria amongst UN5 in SSA?

Four main databases (PubMed, Central, Dimensions and JSTOR) were searched for literature. Medical Subject Heading (MeSH) terms were utilised for the search in PubMed and refined for search in other databases. The search strategy is presented in Table 1, with exclusion and inclusion criteria. The keywords in Table 1 were moved to the MeSH. A planned search strategy in PubMed is presented in Table 2.

**Table 1:** Search strategy for articles on malaria amongst UN5 in SSA

Search strategy item	Search strategy
Databases	PubMed, Central, Dimensions and JSTOR,
Language filter	English Language
Time filter	2000–2022
Spatial filter	“sub-Saharan Africa” OR “Angola” OR “Benin” OR “Botswana” OR “Burkina Faso” OR “Burundi” OR “Cape Verde” OR “Cameroon” OR “Central African republic” OR “Chad” OR “Comoros” OR “Congo” OR “DR, Congo” OR “Cote d’Ivoire” OR “Equatorial Guinea” OR “Eritrea” OR “Eswatini” OR “Ethiopia” OR “Gabon” OR “Gambia Ghana” OR “Guinea” OR “Guinea Bissau” OR “Kenya” OR “Lesotho” OR “Liberia” OR “Madagascar” OR “Malawi” OR “Mali” OR “Mauritania” OR “Mauritius” OR “Mozambique” OR “Namibia” OR “Niger” OR “Nigeria” OR “Rwanda” OR “Sao Tome & Principe” OR “Senegal” OR “Seychelles” OR “Sierra Leon” OR “Somalia” OR “South Africa” OR “South Sudan” OR “Sudan” OR “Tanzania” OR “Togo” OR “Uganda” OR “Zambia” OR “Zimbabwe.”
Keywords	1. “Malaria” OR “Fever” OR “Plasmodium falciparum” OR “Plasmodium malariae” OR “Plasmodium ovale” OR “Plasmodium vivax” 2. “Prevalence” OR “Percentage” OR “Proportion” 3. “Risk factors” OR “determinants” OR “causes.” 4. “Children under five years” OR “Infant” OR “Neonate” OR “Children” OR “Early Childhood” 5. “Interventions” OR “Policies” OR “Strategy” OR “Protection” OR “Health promotion” OR “Health education”
Inclusion criteria	The paper should be: 1. A peer-reviewed or grey literature 2. A published from 2000 and later 3. Conducted in sub-Saharan African countries 4. Published in the English language 5. Conducted on children under five years and 6. On prevalence, risk factors, health education and promotion interventions of malaria or any of these outcomes
Exclusion criteria	The paper should be: 1. Conducted on children above five years 2. Conducted in countries outside sub-Saharan Africa 3. A study published online before the year 2000 4. A report, review, abstract, minutes, commentary, letter to editors, preprint, literature reviews 5. Outside the variables of interest

**Table 2:** Search strategy in PubMed

Search (#)	Search terms
1	Malaria*[MeSH terms] OR Fever* OR Plasmodium falciparum* OR Plasmodium malariae* OR Plasmodium ovale* OR Plasmodium vivax*
2	Children under five years*[MeSH terms] OR Infants* OR Neonates* OR Children* OR Early Childhood*
3	sub-Saharan Africa* OR Angola* OR Benin* OR Botswana* OR Burkina Faso* OR Burundi* OR Cape Verde* OR Cameroon* OR Central African republic* OR Chad* OR Comoros* OR Congo* OR the Democratic Republic of Congo* OR Cote d’Ivoire* OR Equatorial Guinea* OR Eritrea* OR Eswatini* OR Ethiopia* OR Gabon* OR Gambia Ghana* OR Guinea* OR Guinea Bissau* OR Kenya* OR Lesotho* OR Liberia* OR Madagascar* OR Malawi* OR Mali* OR Mauritania* OR Mauritius* OR Mozambique* OR Namibia* OR Niger* OR Nigeria* OR Rwanda* OR Sao Tome & Principe* OR Senegal OR Seychelles* OR Sierra Leon* OR Somalia* OR South Africa* OR South Sudan* OR Sudan* OR Tanzania* OR Togo* OR Uganda* OR Zambia* OR Zimbabwe*
4	#1 AND #2 AND #3
5	Prevalence*[MeSH terms] OR Percentage* OR Proportion*
6	Risk factors*[MeSH terms] OR OR determinants* OR causes*
7	Intervention*[MeSH term] OR Policies* OR Strategies* OR Protection* OR Health promotion* OR Health education*
8	#5 AND #6 AND #7 Limits: 01/01/2000 to 20/07/2022

These MeSH terms were adapted to fit other databases (Central Dimensions and JSTOR). The authors scrutinised the records obtained, and the Mendeley software was used to remove duplicates. The WHO Library, HINARI, Maternal Surveillance and Response Action Network, Google Scholar, Google and institutional repositories of universities in SSA were searched for additional records. Furthermore, reference lists of eligible records were checked for relevant articles.

The last search was done on July 20, 2022. The authors saved articles that met the eligibility criteria in Mendeley software for data charting. The data were independently extracted by P.Y.K. and A.K.A. and reviewed by M.A. and J.O.S. Details that were removed during the data charting process include authors and year, country where the study was conducted, study design, population, sample size, prevalence, risk factors, intervention, diagnostic strategy and funding information. In addition, all authors resolved misunderstandings and discrepancies during the data extraction process during a weekly meeting. The authors consulted a chartered librarian, Dr. Kwame Kodua-Ntim, at the Sam Jonah Library during the search and screening process. In addition, the authors consulted a review and subject experts to ensure the accuracy and depth of data for this scoping review. Finally, all authors reviewed and familiarised themselves with the extracted data and thematic analysis was done, and the results were presented.

## Results

**Fig. 1:**
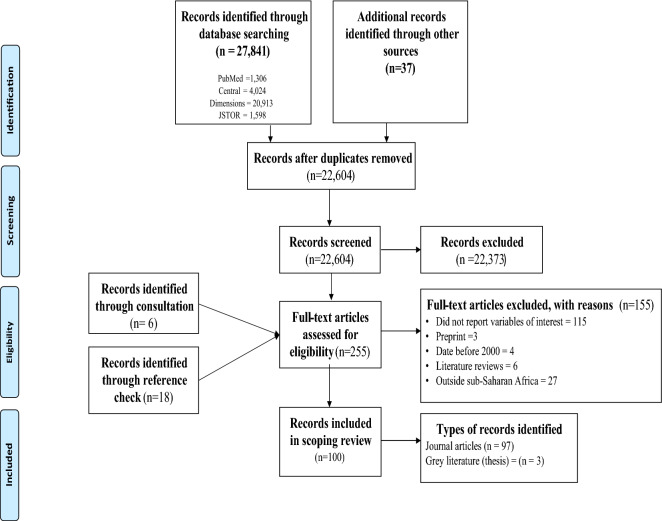
PRISMA flow diagram of search results and record the screening process

The search conducted in the four main databases produced 27,841 records. An additional 37 records were identified through a search conducted in Google, Google Scholar and institutional repositories. After removing duplicates (5274) using the Mendeley software, 22,604 records were available for screening. In addition, 24 papers were retrieved through consultation and reference checking. Furthermore, 22,373 records were excluded because these records did not meet the inclusion criteria. In all, 231 full-text were eligible for further screening. Finally, 100 full-text records were included in the thematic analysis and synthesis (see Fig. 1 for details on the screening process in the PRISMA flow diagram).

### Characteristics of reviewed studies

**Fig. 2:**
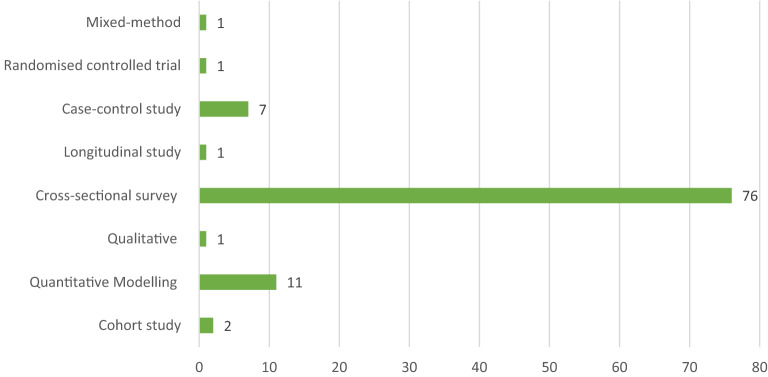
Design of reviewed studies in sub-Saharan Africa

**Fig. 3:**
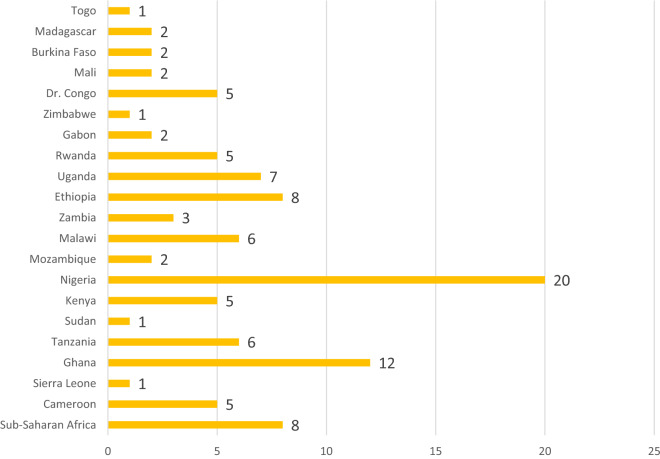
Countries where reviewed studies were conducted in SSA

**Fig. 4:**
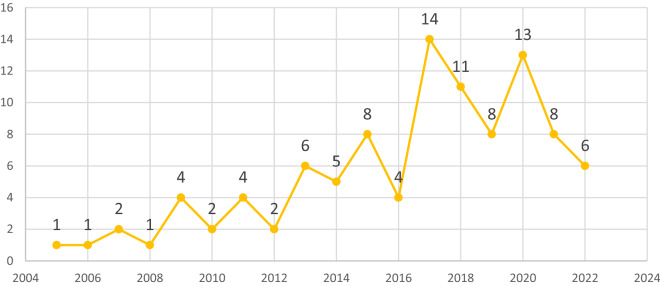
The year reviewed studies were published online

The majority (76) of the reviewed studies used cross-sectional surveys. Furthermore, 40 percent of the included studies were conducted in only three SSA countries; Nigeria (20), Ghana (12), and Ethiopia (8). Again, most of the included studies were published online between 2013 and 2022. See details of the characteristics of the reviewed studies in Figs. 2, 3 and 4.

Twenty-eight (28) of the included studies were funded [10–37]. Regarding diagnostic tools, 21/100 (21%) of the reviewed studies used microscopy [23, 35, 38–56], 16/100 (16%) RDT [11, 21, 24, 33, 57–68] and 2/100 (2%) PCR [15, 27]. In addition, 22 of the included studies used both RDT and microscopy [16, 20, 22, 28, 36, 37, 69–83] and one study used both PCR and microscopy [84] to test for malaria parasites.

### Prevalence of malaria in children under five years in SSA

The reviewed studies reported malaria prevalence between 0.7 percent [20] and 80.3% [49]. Few studies reported a prevalence between 0 and 10 percent [18, 20, 36, 38, 43, 63, 67, 72–74, 77, 85, 86]. Fifty-nine studies reported a prevalence between 11 and 50 percent. Four studies [47, 64, 73, 87] reported a prevalence between 51 and 60 percent, whilst 2 studies reported the highest prevalence of 79.8% [82] and 80.3% [49]. Also, RDT produced a higher prevalence in all studies that used both RDT and microscopy tests for plasmodium parasites analysis [18, 73, 81–83]. This trend is similar to the prevalence reported by a study that used both PCR and microscopy tests [84] (see Additional file 1).

### Risk factors of malaria amongst UN5 in SSA

#### Socio-economic variables

Only one of the reviewed studies reported that in Uganda boys UN5 are at higher risk of malaria infection than girls [70]. In addition, most studies that reported age as a risk factor reported that UN5 who are > 2 years [11, 48, 59, 70, 73, 83, 84, 88, 89] are at higher risk of malaria infection. It is established in the reviewed studies that low household income and poverty [11, 19, 25, 29, 31, 39, 40, 56–58, 68, 70, 77, 87, 90–93] and low maternal education [11, 17, 23, 26, 34, 38–40, 42, 44, 46, 48, 56, 60, 68, 71, 72, 79, 83, 86, 88, 89, 91–97] are risk factors of malaria for UN5 in SSA. In addition, UN5 who reside in rural areas in SSA are more susceptible to malaria infection than those residing in urban areas [11, 26, 42, 57–59, 85, 89, 98]. However, two studies conducted in Burkina Faso [5] and Nigeria [7] reported that UN5 are more vulnerable to malaria infection in urban areas than in rural areas. Moreover, UN5 who walk a long distance to school [65], whose parents are not living together [90] and those that stay outdoor for long [43, 67] are more likely to be diagnosed with malaria.

Several reviewed studies reported poor housing system as a risk factor for malaria infection amongst children UN5 in SSA [26, 31, 42, 65, 79, 82, 83, 89]. For instance, UN5 who live in houses under construction [99] and houses with poor roofing systems [11], are vulnerable to malaria parasites. In addition, UN5 in households without electricity [11, 79], television [41, 57] and toilet [11, 26] are at risk of malaria infection. Reviewed studies further reported that households close (< 5 km) to river or stream [60, 66, 93, 99] and stagnant water [43, 45, 67, 76], a household with poor sanitation [81], unclean water [38, 60, 81] and domestic animals [26, 91] as well as overcrowded households [25, 57, 67, 70, 90, 98] expose UN5 children to malaria infections. Additionally, UN5 in households that do not use insecticide spray [10, 22, 26, 68, 95] or ITN [10, 11, 14, 23, 26, 27, 41, 43, 46, 48, 49, 53, 57, 58, 70, 72, 79, 80, 82, 85, 96, 97, 100–103] are at higher risk of malaria infection.

#### Pre-existing health conditions, infrastructure and health system

Studies show that pre-existing health conditions make UN5 more vulnerable to malaria infections. For instance, UN5 children diagnosed with fever [12, 37, 84, 92], splenomegaly [15], gastro intestinal infection [84], respiratory tract infection [84], low immunity anaemia [90] and previous history of malaria [29, 76] are more likely to suffer malaria infections and complications. In addition, delayed care seeking [30, 87], the use of herbal medicine [87], over-the-counter medications [12] and home management [93] of suspected malaria cases in UN5 are amongst the factors that make children more likely to suffer from malaria complications. In addition, poor roads [87] and long distances to health facilities [94] may prevent mothers of UN5 children from seeking early healthcare. Also, fear of expired drugs might also push mothers to utilise herbal medication [94]. Furthermore, health system issues such as difficult access to health facilities [94], medications [94] and diagnostic test kits [22] are reported as risk factors for malaria in UN5 in SSA.

#### Nutrition and climate change

Nutritional issues, such as malnutrition [21, 72, 80, 99], stunting [55, 84, 89], wasting [24, 72, 84] and lack of micronutrient like iron [15, 39, 57, 77, 103] are reported risk factors that complicate malaria cases amongst UN5. However, the linkage between malnutrition and malaria may need more research attention. Furthermore, little evidence suggests that the changing climate might be a risk factor for malaria in UN5. For instance, low altitude [14, 66, 73] and raining season [69], pose a significant risk of malaria to UN5. Moreover, high altitude has been shown to decrease malaria infection amongst UN5 [41]. Table 3 presents the risk factors reported by authors organised into themes.

**Table 3:** Risk factors of malaria UN5 in SSA

Main theme	Sub theme	Authors
Socio-demographic/economic	Sex (boys)	[70]
	Low education of mothers	[11, 17, 23, 26, 34, 38–40, 42, 44, 46, 48, 56, 60, 68, 71, 72, 79, 83, 86, 88, 89, 91–97]
	Lack of knowledge in malaria prevention	[30, 53]
	Age < 1	[13]
	Age > 1	[80]
	Age < 2	[44]
	Age > 2	[11, 73, 88]
	Age > 3	[48, 59, 70, 84, 89]
	Age > 4	[83]
	Age < 5	[40, 77]
	Poverty/low income	[11, 19, 25, 29, 31, 39, 40, 56–58, 68, 70, 77, 87, 90–93]
	Rural area	[11, 26, 42, 57–59, 85, 89, 98]
	Parents not living together	[90]
	Poor housing system	[26, 31, 42, 65, 79, 82, 83, 89]
	Poor roofing	[11, 71]
	House under construction	[26, 99]
	No electricity	[11, 79]
	No television	[41, 57]
	No toilet	[11, 26]
	Close distance to river < 5 km	[60, 66, 93, 99]
	Close distance to river < 10 km	[65]
	Stagnant water	[43, 45, 67, 76]
	Poor sanitation	[81]
	Unclean water	[38, 60, 81]
	High number of children/ overcrowdings	[25, 57, 67, 70, 90, 98]
	Presence of domestic animals	[26, 91]
	Not using ITN	[10, 11, 14, 23, 26, 27, 41, 43, 46, 48, 49, 53, 57, 58, 70, 72, 79, 80, 82, 85, 96, 97, 100–103]
	Not having ITN	[68, 70, 71, 100]
	Inability to spray rooms	[10, 22, 26, 68, 95]
	Long outdoor stay	[43, 67]
	Long distance to school	[65]
Pre-existing health conditions	Fever	[12, 37, 84, 92]
	Splenomegaly	[15]
	Gastro intestinal infection	[84]
	Respiratory tract infection	[84]
	Low immunity	[49]
	Presence of anaemia	[80]
	Previous malaria status	[29, 76]
	Admission for other health reasons	[32]
Health systems issues	Accessibility	[22, 65, 86, 87, 90–92]
	Long distance to health facilities	[94]
	Insufficient diagnosis test	[22]
	Poor roads	[87]
	Difficult access to medication	[94]
	Fear of expired drugs	[94]
Health seeking behaviour	Delayed healthcare seeking	[30, 87]
	Seasonal malaria chemotherapy	[18]
	Use of herbal or local medicine	[87]
	Over-the-counter medicine	[12]
	Home management	[93]
Climate	Raining season	[69]
	Low temperature < 25	[85]
	Low altitude	[14, 25, 41, 66, 73]
Nutrition	Malnutrition	[21, 72, 80, 99]
	Stunting	[55, 84, 89]
	Wasting	[24, 72, 84]
	Anaemia	[15, 39, 57, 77]

#### Health education and promotion interventions

Health education and promotion is crucial for malaria prevention amongst UN5 in SSA. For instance, it is well **e**stablished in the reviewed studies that health promotion interventions that target increased and effective use of ITN [10, 16, 19, 20, 36, 44, 47, 53–55, 59, 61, 63–65, 70, 71, 86, 88, 92, 97, 103–105] and indoor residual spraying (IRS) [10, 19, 20, 37, 44, 47, 51, 53, 63, 71] have significantly reduced the prevalence of malaria amongst UN5. Furthermore, health promotion activities that ensured malaria vaccination uptake significantly reduced malaria prevalence amongst UN5 in Malawi [36]. Also, giving nutritional supplements to anaemic UN5 to boost their immunity and reduce malaria infections showed encouraging results in Ghana [15].

Moreover, health education strategies that focussed on increasing knowledge amongst mothers and caregivers on treatment of malaria cases amongst UN5 in SSA have successfully reduced malaria complications and mortality amongst UN5 [10, 19, 20, 37, 44, 47, 51, 53, 63, 71]. For example, In Cameroon, educational campaigns targeting mothers and caregivers to access existing free malaria treatments for UN5 led to improved healthcare seeking and decreased hospitalisation for malaria complications amongst UN5 [106]. Again, in Burkina Faso, the implementation of the free healthcare policy was significantly associated with a twofold increase in the number of tested and confirmed malaria cases compared with the period before the policy rollout [22].

Finally, health promotion interventions targeting home-based management have shown a significant reduction in malaria prevalence amongst UN5 in SSA [51, 53, 91, 105]. For example, in Burkina Faso, health promotion intervention through integrated management of malaria in childhood has help mothers rising in far-to reach areas in Burkina Faso to effectively manage malaria cases amongst UN5 [105].

## Discussion

There is still a high prevalence (between 0.07 and 80.3%) of malaria amongst UN5 in SSA. In addition, low or no formal education, poverty or low income and residing in rural areas are risk factors for malaria infection amongst UN5. Furthermore, the poor housing system in SSA and the unavailability of electricity in rural areas and unclean water make UN5 more susceptible to malaria infections. Finally, well-planned health education and education interventions could be successful in reducing malaria risk amongst UN5 in SSA.

### Prevalence of malaria in UN5 in SSA

The high prevalence of UN5 malaria in SSA may be a result of the type and irregular usage of ITNs, the sample used for data collection, the type of diagnostic tests used, climatic conditions, poor treatment of malaria cases amongst UN5 and challenges in implementing existing malaria preventions measures [107]. For instance, studies that used hospitalised samples reported a high prevalence of malaria amongst UN5 [47, 49, 50, 52]. Perhaps, the chances of detecting malaria parasites are always higher for hospital samples since most patients may be showing symptoms and signs of a fever [107], leading to overestimating conditions. It is also established that microscopy produces a lower prevalence than PCR and RDT [18, 81, 83]. Perhaps, may be RDT is highly sensitive and effective in diagnosing latent and active *plasmodium* infections compared to microscopy [108, 109]. Besides, microscopy may produce a high number of false negatives despite its gold standard because of the lack of experienced microscopists and the effect of self-medication which is common in SSA [109]. It is worth noting that chances of false positives with RDT may be due to high rheumatoid factor in samples [109]. These issues may cause discrepancies in malaria prevalence amongst UN5. Finally, the huge gap between ITN ownership and usage may account for the high malaria prevalence amongst UN5 in SSA. For example, a recent meta-analysis in SSA shows an average of 75.8% ownership and 58.3% usage of ITN [110].

### Risk factors of malaria infection in UN5 in SSA

The reported relationship between socio-economic factors like education, area of residence, income level and malaria infections amongst UN5 may be due to the effective prevention, diagnosis and treatment options available in SSA. For instance, education is directly associated with productivity and income or earnings. In addition, education increases knowledge and the ability to access malaria information that promotes health [111]. Perhaps, education and understanding of malaria could translate into the acceptance and practice of malaria prevention interventions that limit infection amongst UN5 in SSA [30].

It is believed that high income earners provide better housing and nutrition that could be essential in malaria prevention amongst UN5. Also, in SSA, there is a large gap in healthcare access and infrastructure between rural and urban residents [112]. Thus, UN5 residing in rural and hard-to-reach areas in SSA may have difficulty accessing effective malaria diagnosis and treatment, vaccination and postnatal care [112, 113], which could lead to complications and death.

Most reviewed studies that reported age showed that UN5 above 2 years are more vulnerable to malaria infection because in SSA, it is highly possible that when most families have a newborn, resources and attention are shifted from older UN5 to the newborn, thereby making older UN5 susceptible to malaria infection [2]. Moreover, children under 2 years are likely to get protection from breastfeeding and increased parental attention, which even protects against undernutrition.

Malnutrition is highly prevalent in SSA amongst UN5 because evidence shows that 39, 8 and 28 percent of UN5 are stunted, wasted and underweight, respectively [29]. Poor nutritional status can lead to immunity suppression, leading to increased risk and poor prognosis of malaria infections amongst UN5 [114]. However, the link between malnutrition and malaria is complex and studies have yielded contradictory results. For instance, in a recent systematic review, anthropometric parameters were unrelated to malaria incidence or parasite density [115]. Hence, more quality studies are needed to highlight this complex association.

### Health education and promotion interventions

The findings show that health education and promotion work to increase the uptake of malaria prevention and control in SSA [116, 117]. This finding indicates that in resource-limited settings such as SSA, health education and promotion of malaria prevention may be effective and cost-friendly when properly planned and executed. For instance, training and mixed communication methods such as interpersonal communication and mass media on malaria prevention and treatment programmes produced effective results [117]. However, limited qualitative studies have effectively evaluated health education and promotion interventions targeting UN5 in SSA. Besides, qualitative studies may provide a deeper understanding of the factors that explain the gap between ITN ownership and usage in SSA.

### Policy implications

This review has shown that children from low socio-economic backgrounds may be at greater risk of malaria infection than their counterparts. Meanwhile, existing malaria preventions, including treatment, ignores people’s socio-economic background and focus mainly on the distribution of ITN, IRS, larval source management, diagnosis and treatment of malaria cases [111, 116–118]. Therefore, the government and policymakers in SSA need to consider improving the socio-economic status of its people as an additional measure to eradicate malaria amongst children effectively. However, there is a variation in socio-economic factors that predispose UN5 to malaria infections in SSA [119]. As a result, governments and policymakers need to consider multiple policies rather than relying on a single one to reduce or eliminate health disparities to achieve the required results in reducing the malaria burden amongst UN5 in SSA.

Furthermore, there is a need to bridge the equity and inequality gap in healthcare access and infrastructure that exist between rural and urban areas in SSA. Reducing or eliminating this gap means providing accessible, affordable, quality healthcare to rural dwellers. Thus, governments, non-governmental organisations and policymakers should contribute to making malaria prevention, including treatment and diagnosis services, financially and geographically accessible to people in SSA. This intervention should not ignore mothers living in urban slums or poor urban areas and those who live on the street and in displaced and violent communities in SSA.

Reviewed studies indicated that malaria prevention interventions that combine training and interpersonal communication with media, community mobilisation and involvement in interventions effectively reduce malaria infection amongst UN5 in SSA [117]. Hence, community-based malaria prevention and control interventions should go beyond knowledge enrichment to influencing behavioural changes that may significantly reduce the malaria burden on UN5 in SSA.

### Recommendations for future research

There is a need for further exploration of the linkage between malnutrition and malaria infection using high-quality studies such as randomised controlled trials and other epidemiological methods like cohort and longitudinal studies. Furthermore, future studies should focus on qualitative designs that evaluate malaria interventions in SSA that target UN5 for deeper knowledge and understanding of what works and what needs improvement. The linkage between climate change and malaria amongst UN5 in SSA may need quality research attention.

### Limitations

This review used only studies published in the English language, which might limit the number of studies that could have enriched the findings of this review. Moreover, most reviewed studies were cross-sectional surveys, so the causal relationship between socio-economic factors and malaria may not be fully established. However, authors retrieved 100 studies from over 20 African countries for this review to help map relevant evidence and provide recommendations for policy actions.

## Conclusion

There is still a high prevalence of malaria amongst UN5 in SSA. In addition, socio-economic factors such as low or no formal education, poverty or low income and residing in rural areas are risk factors for malaria infection amongst UN5. However, evidence on age and malnutrition as risk factors for malaria in UN5 is inconsistent and inconclusive. Furthermore, the poor housing system in SSA and the unavailability of electricity in rural areas and unclean water make UN5 more susceptible to malaria infections. Finally, well-planned health education and education interventions could reduce malaria risk amongst UN5 in SSA. Hence, policies that support access to malaria diagnosis, treatment and prevention tools for low income groups and maximise individual and organisational commitment to strengthening malaria control activities may effectively reduce the malaria burden amongst UN5 in SSA.

## Supplementary Information

**Additional file 1: Table S1.** Data extraction for included studies.

## Data Availability

All resources used in this study are available online and the authors will make them available according to their respective copyright and access policies.
