# Supplementary material for: Malaria amongst children under five in sub-Saharan Africa: a scoping review of prevalence, risk factors and preventive interventions
Source: Eur J Med Res. 2023 Feb 17;28:80. doi: 10.1186/s40001-023-01046-1 (PMC9936673; doi:10.1186/s40001-023-01046-1)
Supplement: Supplementary file 1 — Additional file 1: Table S1. Data extraction for included studies. [file 40001_2023_1046_MOESM1_ESM.docx]

**Table S1: Data extraction for included studies**

| **Author and country** | **Purpose of the study** | **Design** | **Population** | **Sample size** | **Prevalence** | **Risk factors** | **Intervention/ policies** | **Funding** | **Diagnostic**  **tool** |
| --- | --- | --- | --- | --- | --- | --- | --- | --- | --- |
| [10]  Sub-Saharan Africa | To quantify the effects of malaria interventions at national and subnational levels. | Quantitative (Modelling) | Children under-five |  |  | Children who were not sleeping under insecticide treated net (ITN). Inability to spray rooms with insecticide sprays | Supply of insecticides treated nets, indoor residual spraying at the local level (Spraying with Chemicals) led to reduction in malaria cases. | Funded |  |
| [11]  Malawi | To investigate the prevalence and factors associated with malaria in children under five years old. | Cross-sectional survey | Under five | 2,724 | 37% | Children residing in rural areas, low-income level of families, mother’s educational level, children >2years, children with anaemia, children who do not have toilet facilities at home, homes with no electricity, main roof material which are not well fixed and children not sleeping under ITN are risk factors. |  | Funded | RDT |
| [12]  Kenya | To assess childhood and adult patterns of over-the-counter medicine use to inform national medicine retailer programmes | Cross sectional survey | Children under-five | 11,505 |  | Children who reported with fever, fever treated with over-the-counter medicines, rapid malaria test positive | Improved over the Counter anti malaria medicine to high risk groups, communication strategy on adults as well as children dosage to effectively use Over-the-ounter drugs | Funded |  |
| [13]  Mozambique | To characterize the malaria transmission intensities and to estimate the proportion of fever cases attributable to malaria infections in order to establish the malaria case definition. | Quantitative  (modelling) | Children under-five | 6,641 | <1 year, 43.5%; 1 – 5 years, 39.6% | Children who are <1 year |  | Funded |  |
| [14]  Zambia | To estimate an empirical high-resolution parasitological risk map in the country and to assess the relation between malaria interventions and parasitaemia risk after adjusting for environmental and socio-economic confounders. | Quantitative modelling | Children under-five | 2,364 | 26.4% | Low altitude and non-use of ITN |  | Funded |  |
| [15]  Ghana | To determine the association that exists between chronic undernutrition and PCR-confirmed cases of asymptomatic malaria | Cross sectional survey | Children under-five | 214 | 31.8% (68) | Children who experience anaemia and children who have splenomegaly. | Continuous use of nutritional component. Exceptionally, components that address malaria which led to reduction in malaria cases | Funded | PCR |
| [16]  Ethiopia | To determine parasite and anaemia prevalence in the population at risk and to assess coverage, use and access to scaled-up malaria prevention and control interventions. | Cross sectional survey | Under five | 5,243 |  |  | Involvement of community members to increase use of ITN. This led to reduction in malaria cases | Funded | Microscopy  RDT |
| [17]  Nigeria | To determine baseline information on the perceptions of the causes and treatment of fevers suspected to be malaria in children less than five years in Rivers State | Cross sectional survey | Mothers and caregivers | 811 |  | No formal education of mothers | Community-directed management options that utilizes trained community-based volunteers were introduced.  There was increase in knowledge on occurrence of malaria and treatment among mothers of under five children | Funded |  |
| [18]  2020  Nigeria | To assess the impacts of SMC on malaria burden among subjects aged 3–59 months in Borno State, Nigeria. | Cross sectional survey | Under five | 399 | Microscopy, 10.3%  RDT, 15.9% | Children who were not on SMC were at risk | There was supply of Seasonal Malaria Chemoprevention (SMC) which made prevalence of the malaria to reduce to 10.3% among children who used SMC compared to 15.9% among non-use kids. | Funded | RDT Microscopy |
| [19]  Tanzania | To examine the prevalence of malaria and the associated factors among the under-five year‘s children in Muleba District. | Cross sectional survey | Under five | 391 | 26.3% | Poverty level of households | The use of long-lasting Insecticidal Nets (LLIN), Artemisinin-based Combination therapy (ACT), Indoor Residual Spraying of insecticide (IRS), and Intermittent Preventive Treatment in Pregnancy (IPT). | Funded |  |
| [20]  Zambia | To reduce malaria incidence by 75% and under-five mortality due to malaria by 20% by the year 2010" | Cross-sectional survey | Under five | 1,378 | 0.7% |  | The use of long-lasting insecticide-treated nets and indoor residual spraying, the use of artemisinin-based combination therapies (ACT) for the treatment of uncomplicated malaria, improving diagnostic capacity (both microscopy and rapid diagnostic tests), use of intermittent presumptive treatment for pregnant women, research, monitoring and evaluation, and behaviour change communication. Financial barriers to access have been removed by providing free malaria prevention and treatment services.  The above led to reduction of malaria cases | funded | RDT  Microscopy |
| [21]  Cameroon | To investigate the relationship between malaria, anaemia, nutritional and socio-economic status amongst under—ten children living in six localities within two health districts in the North Region of Cameroon | Cross sectional survey |  | 182 | 32.9% | Malnutrition |  | Funded | RDT |
| [22]  Burkina Faso | This study aimed at determining the magnitude of malaria and associated factors among febrile children under 5 years old in Arba Minch “Zuria” dis The present study aims at filling this gap by assessing the effect of this policy in children under five with a focus on the induced spatial and temporal changes in malaria morbidity. tri The present study aims at filling this gap by assessing the effect of this policy in children under five with a focus on the induced spatial and temporal changes in malaria morbidity. ct  To assess the effect of free of charge healthcare policy in children under five with a focus on the induced spatial and temporal changes in malaria morbidity. | Quantitative modelling | Under five | 12,874 |  | Low indoor residual spraying  Inaccessibility to healthcare  Insufficient diagnosis test | Implementation of the free healthcare policy was significantly associated with a two-fold increase in the number of tested and confirmed malaria cases compared with the period before the policy rollout. | Funded | Microscopy  RDT |
| [23]  Cameroon | To determine the predictors of malaria prevalence and coverage of ITBNs among under-five children in the Buea Health District. | Cross sectional survey | Children under-five | 391 | 13.04% | Low educational level of parents and Children non-use of ITN. |  | Yes | Microscopy |
| [24]  Tanzania | To assess the situation of malnutrition in under-five children and how it interacts with malaria. | Cross sectional survey | Children under-five | 2,243 | 14% | Wasted |  | Funded | RDT |
| [25]  Rwanda | To determine if they are associated with ITN non-use  among children under 5 years of age in Rwanda. | Cross-sectional survey | Men and women with children under-five | 6,173 |  | Household with more than five members, employed mother and lower altitude. |  | Yes |  |
| [26]  SSA | To identify significant household factors influencing the risk of malaria  parasitaemia among children under the age of five years. | Quantitative modelling | Children under-five |  |  | Poor households, household constructed with natural and rudimentary materials, unimproved toilet facilities, Non-use of IRS or ITN and children of mother with no or primary education, households who kept cattle and residing in rural areas are risk factors. |  | Yes | RDT and Microscopy |
| [27]  Dr Congo | To evaluate the effect of individual versus community-level use to prevent malaria among children under the age of 5.  To evaluate the effect of individual versus community-level use to prevent malaria among children under the age of 5 | Cross-sectional survey | Children under-five | 5,857 | 37.4% | Non-use of ITN |  | Yes | PCR |
| [28]  Ethiopia | To evaluate changes in malaria control policy.  To evaluate changes in malaria control policy | Quantitative  (Modelling) | Children under five years |  |  | Construction of dams increases malaria transmission in the valleys of highland areas, population migration and treatment-seeking behaviour may have contributed to the high level of API observed in these highlands. |  | Funded | RDTs and  Microscopy |
| [29]  Ethiopia | To assess the relationship between malaria and malnutrition among under five children in an area with a high degree of malaria transmission. | Case-Control study | Children under five years | 356 | 48% | Low family income, previous Malaria Status |  | Funded |  |
| [30]  Nigeria | To describe rural–urban disparity in fever prevalence, mothers’ malaria knowledge, and associated factors with delayed care-seeking for fever in U5 and testing fever for malaria before anti-malarial drugs.  To describe rural–urban disparity in fever prevalence, mothers’ malaria knowledge, and associated factors with delayed care-seeking for fever in U5 and testing fever for malaria before anti-malarial drugs. | Cross sectional survey | Children under five years | 630 | 26% | Delayed care seeking for fever, perception of malaria as not a major health problem in the community, poor knowledge of mosquitoes’ feeding time |  | Funded | RDT & Microscopy |
| [31]  SSA | To investigate the association between malaria and :  (i) cleaner fuel usage; (ii) wood compared to charcoal fuel;  and, (iii)household cooking location, among children aged under 5 years in sub-Saharan Africa (SSA) | Cross sectional survey | Children under five years | 85,263 |  | Use of wood and charcoal fuel are risk factors, longer cooking times, household construction characteristics (eaves space, wall type) and poor living conditions of the household |  | Funded | Microscopy and RDTs |
| [32]  Mali | To assess the impact of seasonal malaria chemoprevention (SMC) on hospitalizations and deaths of children under 5 years of age during the second year of implementation of SMC.  To assess the impact of seasonal malaria chemoprevention (SMC) on hospitalizations and deaths of children under 5 years of age during the second year of implementation of SMC. | Case-control study | Children under five | 6,638 |  | Children on admission for other health reasons are at risk. |  | Funded |  |
| [33]  Sub - Saharan Africa | To assess the validity of malaria diagnosis and treatment coverage indicators collected during household surveys  To assess the validity of malaria diagnosis and treatment coverage indicators collected during household surveys | Case-control study | Children under five | 400 |  | Having higher household wealth and being able to read |  | Funded | RDT |
| [34]  Nigeria | To assess caregivers’ knowledge about Long lasting insecticidal nets, utilization of LLIN and factors influencing LLIN use among under five  To assess caregivers’ knowledge about Long lasting insecticidal nets, utilization of LLIN and factors influencing LLIN use among under five | Cross Sectional Survey | Children under five years | 1020 |  | Low level of Knowledge and education on ITN by respondents. |  | Funded |  |
| [35]  Sub Saharan Africa | To evaluate estimates of *Pf*PR using microscopy, over a period of 3 years in sites from several sub-Saharan African countries,  To evaluate estimates of *Pf*PR using microscopy, over a period of 3 years in sites from several sub-Saharan African countries | Cross Sectional Survey | Children under five | 21,611 |  | Sleeping under a bed net  Participants who reported having received an anti-malarial treatment in the previous 14 days had lower risks of malaria |  | Funded | Microscopy |
| [36]  Malawi | To investigate the effect of community-level of ITN use against malaria transmission outside of a bed net clinical trial setting  To investigate the effect of community-level of ITN use against malaria transmission outside of a bed net clinical trial setting | Cross Sectional Survey | Children under five years | 1,200 | 5.6% |  | Distribution of ITN, community vaccination | Funded | RDT & Microscopy |
| [37]  Mozambique | To study the prevalence and factors associated with malaria  To study the prevalence and factors associated with malaria | Cross Sectional Survey | Children under five | 2540 | 13% | Fever in children | There was an indoor residual spraying. | Funded | RDT Microscopy |
| [38]  Rwanda | To measure the prevalence of malaria parasitaemia, anaemia and under-nutrition among preschool age children.  To measure the prevalence of malaria parasitaemia, anaemia and under-nutrition among preschool age children. | Cross Sectional survey | Children under five years | 3182 | 10.25% | Older children are at risk.  Lack of educations in the homes about malaria, open water sources serving as potential mosquito breeding sites. |  |  | Microscopy |
| [39]  Uganda | To investigate factors associated with malaria prevalence and its relationship with anaemia.  To investigate factors associated with malaria prevalence and its relationship with anaemia. | Cross-sectional survey | Children under five years | 4930 |  | Children with anaemia, low -level of education of mothers and households with low income. |  | Not Funded | Microscopy |
| [40]  Togo | To explore further such regional differences in malaria prevalence and to determine associated risk factors. | Cross Sectional survey | Children under five years | 171 | 31.6% | Children <5years, low  education level of mothers, low household income. |  | Not Funded | Microscopy |
| [41]  Ethiopia | To assess factors associated with malaria among under-five children in Ethiopia  To assess factors associated with malaria among under-five children in Ethiopia | Cross-sectional survey | Children under-five | 8,301 |  | Sleeping under ITN, having two or more ITN for the household, low altitude, availability of radio and television are risk factors. |  | No | Microscopy |
| [42]  Nigeria | To determine the current sociodemographic determinants of malaria in ill children visiting a tertiary hospital which offers primary, secondary and tertiary levels of care to its catchment area | Cross sectional survey | Children under-five | 203 | 16.7% | Mothers of children with lower education, poor households and residing in rural areas. |  |  | Microscopy |
| [43]  Ethiopia | To determine the prevalence and associated factors of malaria in children under five years in low transmission area | Cross sectional survey | Children under-five | 525 | 8.7% | Children living in households with stagnant water, children who do not sleep under treated mosquito net and children who stay outside at night |  |  | Microscopy |
| [44]  Dr. Congo | To identify malaria socioeconomic predictors among children aged 6–59 months | Cross sectional survey | Children under-five | 8,547 | 25% | Children <2 years, mothers with low educational level and children living with single mothers | Distribution of Insecticide-Treated Nets (ITNs), promotion of indoor residual spraying, promotion and implementation of intermittent preventive treatment in pregnancy, promotion of rapid diagnostic tests, and implementation of community and mother case management with artemisinin-based combination therapies |  | Microscopy |
| [45]  South Ethiopia | This study aimed at determining the magnitude of malaria and associated factors among febrile children under 5 years old in Arba Minch “Zuria” distriThis study aimed at determining the magnitude of malaria and associated factors among febrile children under 5 years old in Arba Minch “Zuria” districtct  To determine the magnitude of malaria and associated factors among febrile children under 5 years old in Arba Minch “Zuria” district | Cross sectional survey | Children under-five | 271 | 22.1% | Children who were not sleeping under ITN and stagnant water around households which leads to breeding of mosquitoes |  |  | microscopy |
| [46]  Nigeria | This study was conducted to determine the occurrence of malaria among children underfive years | Cross sectional survey | Children under-five | 220 | 14.5% | Mothers who lack knowledge on preventive measures are at risk. |  |  | microscopy |
| [47]  Uganda | To determine the prevalence, presentation and treatment outcomes of malaria and anemia among children in two hospitals in Rakai, Uganda. | A cohort study | Under five hospitalized children | 2,471 | 54.6% |  | There was use of artemisinin-based combination therapy, distribution of insecticide treated bed nets, intermediate preventive therapy, indoor residual spraying, improve monitoring of effectiveness of current control strategies. |  | Microscopy |
| [48] | To compare the prevalence and factors associated with malaria parasitemia among children under the age of five. | Cross sectional survey | Children under-five | 4,040 | 33% | Children who do not sleep under treated mosquito nets children who were forty-eight month of age. Children with illiterate mothers |  |  | Microscopy |
| [49]  Nigeria | To determine the prevalence of malaria among the under-five Nigerian children in a resource-poor setting of a rural hospital in Eastern Nigeria | Cross sectional survey | Mothers and caregivers of Children under-five | 244 | 80.3% | Low usage of ITN, children with low immunity, children between age 3 to 5 were not frequently sleeping in the nets. |  |  | Microscopy |
| [50]  Gabon | To examined the prognostic indicators of severe falciparum malaria in Gabonese children. | Cross sectional survey | Under five | 8,036 | 39.3% |  |  |  | Microscopy |
| [51]  Rwanda | To determine the prevalence of malaria parasites and risk factors associated with malaria infection among children under-five years in Huye district, Rwanda. | Cross sectional survey | Children under five | 222 | 12.2% | Children <1 year, children who were not sleeping under ITN | Long lasting indoors residual spraying, effective case management have resulted in significant decline in malaria incident |  | Microscopy |
| [52]  Nigeria | To determine the prevalence of malaria parasitaemia among the under -five aged children and the effect of various determinants. | Cross sectional survey | Hospitalised children under-five | 433 | 27.7% |  |  |  | Microscopy |
| [53]  Nigeria | To evaluate the association between LLIN distribution campaigns and child malaria in Nigeria. | Cross sectional survey | Under five | 4,082 | 42% | Lack of maternal knowledge about malaria prevention, children who were not using ITN and children in areas with no malaria campaign | Long lasting insecticides net distributions.  There was an increased indoor residual spraying, and environmental management to decrease mosquito breeding places |  | Microscopy |
| [54]  Gabon | To estimate the clinical burden of malaria among febrile children aged less than 11 years, before and after six-year of deployment of malaria control strategies in different areas of Gabon | Cross-sectional survey | Under five | 13,212 | 29.5% |  | Deployment of treated mosquito nets to households  Artemisinin-based combination therapy (ACT)  The intervention led to decrease in malaria among children under five years |  | Microscopy |
| [55]  Uganda | To prove or disprove if malnutrition is associated with risk of malaria. | A cohort study | Under five | 358 |  | Mild and severe stunted children are at risk of malaria. | Children were provided with insecticide-treated bed nets at enrolment and daily trimethoprim-sulphathiazole prophylaxis (TS) was prescribed for HIV-exposed breastfeeding and HIV-infected children.  After the intervention, it was detected that malnutrition leads to increase in malaria. |  | Microscopy |
| [56]  Kenya | To compare the discrepancy in malaria and anaemia burdens between symptomatic diagnosed patients with those diagnosed through the laboratory. | Longitudinal study | Under five | 887 | 35.87% average prevalence. | Poverty and ignorance about the disease. |  |  | Microscopy |
| [57]  Ghana | To use machine learning techniques to identify variables to build the best fitting predictive model of malaria prevalence in Ghana | Cross sectional survey | Under five | 2,867 | 25.04% | Not sleeping under ITN, households with low or no income, high number of children under 5 in a household, anaemia, children living in rural areas  and households without television are risk factors. |  |  | RDT |
| [58]  Tanzania | To determine prevalence of malaria and social demographic factors related with children under-five in Tanzania. | Cross sectional survey | Under five | 9,322 | 11.97% | Children>4 years who do not always use ITN, children living in rural areas, families who do not use ITN and low-income level of parents are risk factors. |  |  | RDT |
| [59]  Uganda | To assess intervention effects on malaria prevalence in Uganda among children less than 5 years. | Quantitative  (modelling) | Under five | 4,939 | 19.5% | Malaria was higher in rural areas, children who advance in age >3 years, low socio-economic factors. | ITN possession and usage led to reduction of malaria and there was also indoor spraying with insecticides. |  | RDT |
| [60]  Malawi | To compare the risk factors of malaria experienced by children under the age of five from Zomba district, who reside in lakeshore and highland areas. | Case-control study | Under five | 765 |  | Households closer (<1km) to stagnant water, households with borehole or unprotected well and not attending health talks on malaria preventions programs. |  |  | RDT |
| [61]  Tanzania | To evaluate the extent to which malaria control intensification programme led to reduction in all cause -under five child mortality | Cross-sectional survey | Children under-five | 16,926 |  |  | Distribution of ITN and public education on malaria prevention. There is 45% reduction in under-five child mortality and50% reduction in SAP following malaria intervention scale-up is a major public health achievement for Tanzania. |  | RDT |
| [62]  Kenya | To evaluate the impact of providing subsidized artemether–lumefantrine (AL) through retail providers on the coverage of prompt, effective antimalarial treatment for febrile children aged 3–59 months. | Randomised controlled trial | Children under-five | 2,662 | Control, 27%  Intervention  32.4% |  | Provision of subsidize packs of pediatric ACT to retail outlets, training of retailers and creating community awareness |  | RDT |
| [63]  Tanzania | To evaluate the protective effect of pyrethroid IRS and ITNs in relation to risk factors for malaria. | Cross sectional survey | Children under-five **(1-5)** | 3278 | Survey 1, 10.4%  Survey 2, 23.7% |  | Indoor residual spraying with mosquito sprays and distribution of ITN |  | RDT |
| [64]  Nigeria | To assess the impact of intervention strategies that integrated patent medicine vendors into community case management of childhood diseases, improved access to artemisinin combination therapy (ACT) and distributed bed nets to households. | Case-control study | Under five | 5814 | Pre intervention  56.6%  Post intervention  42.5% | Mothers with malaria related education from the intervention. | Upgraded health facilities for better provision of treatment for malaria, train community base management of childhood illness,  There was also supply of commodities such as Artemisinin based Combination Therapy distribution of ITNs to families. These resulted in reducing the prevalence and the likelihood of childhood malaria fever. |  | RDT |
| [65]  Rwanda | To assess the prevalence of malaria among children aged six months to 14 years old in Rwanda and to identify the factors associated with malaria in this age group. | Cross sectional survey | Children under-five | 2,407 | 14.5% | Lack sufficient access to healthcare facilities, poor housing conditions, children of school going age get infected while travelling to and from school, travelling through forest or agriculture plantations. | There was distribution of ITN |  | RDT |
| [66]  Nigeria | To evaluate important risk factors of under-5 malaria prevalence | Cross-sectional survey | Children under five | 6,025 | 45.1% | Short distance to water bodies and low altitude |  |  | RDT |
| [67]  Ethiopia | To assess the Knowledge on malaria prevalence and associated factors among under-five children. | Cross sectional survey | Under five | 356 | 3.9% | Stagnant water near houses leads to breeding of mosquitoes, large number of under five children >5 and outdoor stay at night |  |  | RDT |
| [69]  Cameroon | To determine the epidemiological and clinical aspects, and outcome of children with severe malaria | Quantitative  (modelling) | Children under five | 323 | 26.15% | Rainy season |  |  | RDT and microscopy |
| [70]  Uganda | To assess the change in the prevalence of malaria infection among children below five years of age between 2004 and 2010 | Cross sectional survey | Under five | 2,847 | 2004  43%  2010  23% | Children who are >3 years and male boys, children who do not use bed net during the night, household with family size, wealth and economic status of household (poverty), education level of the head of the household, households who do not own ITN | Introduction ITN to decrease in malaria cases in communities. |  | Microscopic  RDT |
| [71]  Tanzania | To find out malaria and anemia situation in under-five children and its influencing socioeconomic factors. | Cross sectional survey | Children under-five | 2,340 | 15.9% | No formal education, household number of people, household number of under-five, not having a bed net, thatched roof, sand/soil floor and low socio-economic status. | Distribution and use of ITN  Indoor residual spraying |  | RDT  microscopy |
| [72]  Ethiopia | To assess whether malnutrition is associated with malaria among under-f Shikur i Shikur ve children.  To o assess whether malnutrition is associated with malaria among under-five children. | Case-control study | Children under-five | 428 | Case, 17.8%  Control 9.3% | Severe wasting, underweight, sleeping under LLIN and care-takers who had no education are risk factors |  |  | Microscopy and RDT |
| [73]  Ghana | To assess the prevalence of malaria among children less than five years residing in High-Altitude and Low-Altitude rural communities.  To assess the prevalence of malaria among children less than five years residing in High-Altitude and Low-Altitude rural communities in the Hohoe municipality. | Cross-sectional survey | Children under-five | 325 | Low altitude; RDT, 56.9%; Microscopy, 41.4%  High altitude; RDT 19.9%; Microscopy 3.3%. | Children >2 years, low altitude, mild anaemia and low Hb. |  |  | RDT and Microscopy |
| [74]  Ghana | To assess the prevalence of malaria, anemia, ownership and use of LLINs at the end of the low transmission (pre-rainy season) and high transmission (post-rainy season) seasons in 2015  To assess the prevalence of malaria, anemia, ownership and use of LLINs at the end of the low transmission (pre-rainy season) and high transmission (post-rainy season) seasons in 2015 | Cross Sectional Survey | Children under five years | 1,648 | 8.6% | Rainy Season |  |  | RDT  Microscopy |
| [75]  Sub-Saharan  Africa | **To estimate the association of malaria parasitaemia, anemia, and malariaanemia comorbidity with all-cause under-five mortality and evaluated the potential of malaria-anemia comorbidity prevalence to quantify malaria-related deaths in sub-Saharan Africa**  To estimate the association of malaria parasitemia, anemia, and malaria anemia comorbidity with all-cause under-five mortality and evaluated the potential of malaria-anemia comorbidity prevalence to quantify malaria-related deaths in sub-Saharan Africa | Quantitative  (Modelling) | Children under-five years |  | 31.1% |  |  |  | Microscopy  (RDT) |
| [76]  Ghana | To assess the burden of malaria and caregivers’ health‑ seeking behaviour for children under five.  To assess the burden of malaria and caregivers’ health‑seeking behaviour for children under five. | Cross-sectional survey | Children under five years | 372 | 34.1% | Stagnant residual water from mining activities in close proximity <25 m) to homes in communities, inadequate control and experience of younger caregivers in handling issues of malaria, child having a history of frequent malaria |  |  | RDT & Microscopy |
| [77]  Nigeria | To assess the relationship between housing type and malaria prevalence among under 5 children in Nigeria.  To assess the relationship between housing type and malaria prevalence among under 5 children in Nigeria. | Cross sectional survey | Children  under five | 6991 | 3.85% | Children living with severe anemia, children <5years, low level income of parents. |  |  | RDTs and Microscopy |
| [79]  Uganda | To investigate the relationship between the malaria status of children under the age of five years.  To investigate the relationship between the malaria status of children under the age of five years. | Cross-sectional survey | Children under five years | 4939 | 19.7% | Non-availability of electricity in the household, Poor /unimproved flooring quality was associated with a higher risk of malaria, children who do not sleep under ITN, low educational level of caregiver is a risk factor. |  |  | RDT Microscopy |
| [80]  DR Congo | The objective of this study was to assess PI prevalence and its relationship with known morbidity factors in a vulnerable but asymptomatic stratum of the population.  The objective of this study was to assess PI prevalence and its relationship with known morbidity factors in a vulnerable but asymptomatic stratum of the population. | Cross-sectional survey | Children under five years | 700 |  | Children older than <1 year presence of anemia, Chronic malnutrition and he non-use of insecticide-treated nets increases the risks of malaria |  |  | Microscopy & RDT |
| [81]  SSA | To analyze whether improved drinking water and sanitation conditions were associated with a decreased risk of malaria infection | Cross sectional survey | Children under five | 247,440 | Microscopy, 18.8%; RDT, 24.2% | Unhygienic water and poor sanitation are risk factor. |  |  | RDT and microscopy |
| [82]  SSA | To Test the hypothesis that the odds of malaria infection are lower in modern, improved housing compared to traditional housing in sub-Saharan Africa (SSA) | Cross sectional survey | Under five | 284,532 | Microscopy  Modern house (45.5%), traditional house (70.6%)  RDT  Modern houses (61.2%), traditional house (79.8%) | Poor housing units and children who were not sleeping in ITN. |  |  | RDT  Microscopy |
| [83]  Sierra Leone | To examine the relationship between malaria status in under-five children and household demographic, socioeconomic, and environmental risk factors potentially associated with the disease and to compare the test performance of rapid diagnostic test kit.  to examine the relationship between malaria status in under-five children and household demographic, socioeconomic, and environmental risk factors potentially associated with the disease and to compare the test performance of rapid diagnostic test kit. | Cross-sectional survey | Children under-five | 6,720 | RDT 52.67%; Microscopy 40.05% | Children >4 years, poor household income, children whose mothers have primary or no education, household with unimproved wall materials. |  |  | RDT and Microscopy |
| [85]  Kenya | To design effective strategy for the control of malaria among children | Quantitative modelling | Children under five | 20,570 | 7.8% | Children living in rural areas, temperature under 25, long distance to main water bodies, children who are >3 and non-use of ITN are risk factors. | Proposal and implementation of annual surveys of malaria infection prevalence leading to reduction in malaria cases |  |  |
| [86]  Madagascar | To examine the role of socioeconomic inequalities by broadly predicting malaria knowledge and use of preventive technology among women.  To examine the role of socioeconomic inequalities by broadly predicting malaria knowledge and use of preventive technology among women. | Cross-sectional survey | Children under five years | 7644 | 7.8% | Low educational level of the mother, less number of healthcare facilities in the community | Distribution of ITN, free or low-cost treatment, routine surveillance, and access to effective antimalarial drugs |  |  |
| [87]  Nigeria | To investigate determinants of delay in seeking early and appropriate malaria treatment for children o -5 years. | Cross sectional survey | Under five | 738 |  | Delay in assessing healthcare, poor roads impeding hospital accessibility, believe in local medicines and low income |  |  |  |
| [88]  Nigeria | To investigated the factors associated with utilisation of long-lasting insecticide treated net (LLIN) and parasitaemia among under-five children in 13 States with high malaria burden | Cross sectional survey | Children under five | 2,844 | 26.4% | Children >2 years and children of uneducated women | Introduction and use of LLINs |  |  |
| [89]  Nigeria | To evaluate the association between stunting, underweight, wasting, and malaria among under-five Nigerian children while assessing the effects of sociodemographic factors  To evaluate the association between stunting, underweight, wasting, and malaria among under-five Nigerian children while assessing the effects of sociodemographic factors | Cross-sectional survey | Children under-five | 12,996 | 22.6% | Children >3 years, rural residents, stunted, poor households, and children with no formal education are risk factors. |  |  |  |
| [90]  Nigeria | To determine proximate family biosocial variable associated with severe malaria among under-five children in a resource-poor setting of a rural hospital in Eastern Nigeria | Cross-sectional survey | Children under-five | 220 | 31.8% | Family size >4, low social class, nonliving together of parents and poor access to health facilities. |  |  |  |
| [91]  Sudan | To identify the basis on which fever was recognized and classified and exploring factors involved in selection of different treatment options. | Mixed-method study | Children under-five | 96 |  | Accessibility to health facilities, low educational level of mothers low income level of households, presence of domestic animals are risk factors | Home base management strategies were implemented to encourage and improve good treatment options |  |  |
| [92]  Nigeria | To assess the progress made in ITN ownership use among pregnant women and children under five years | Cross sectional survey | Children under-five and mothers | 5,588 | 7.1% | Fever, presence of health facility in the community, caregiver's education, residence, and wealth index by caregiver's education; while religion, presence of health facility and wealth index by caregiver's education predicted the use of ITN | There was 11% prevalence of ITN usage among children under-five years. |  |  |
| [93]  Zambia | To determine the barriers to prompt malaria treatment among children under five years of age with malaria in Mpika district | Cross sectional survey | Caretakers of under-five | 380 | 13.9% | Far distance (>5km) to health facilities, inadequate household income, lack of community health education and self-initiated treatment at home. |  |  |  |
| [94]  Malawi | To assessed socio-cultural factors associated with delayed treatment of children with fever | Qualitative  Study | Mothers and healthcare providers | 197 |  | Inadequate knowledge on the correct dosage of malaria drugs, poor access to anti malaria drugs at community level, fear of giving expired drugs and long distance to health facilities | Education to facilitate prompt and appropriate health seeking behaviour, knowledge that will change or address the prevailing local believes about causes of fever. |  |  |
| [95]  Ghana | To analyze and map malaria risk in children under 5 years old, with the ultimate goal of identifying areas where control efforts can be targeted.  To analyze and map malaria risk in children under 5 years old, with the ultimate goal of identifying areas where control efforts can be targeted. | Quantitative  (Modelling) | Children under five | 2,537 | 22.1% | Age, IRS use, social economic status and mother’s education level. |  |  |  |
| [96]  Ghana | To examine the correlates of the high under-five mortality among children in the northern part of Ghana, with emphasis on the usage of ITN, as recommended by the World Health Organization | Cross-sectional survey | Children under five | 3,839 |  | Children not sleeping under ITN were at risk, children not sleeping under untreated bed nets, poverty, low level or no education on the part of mothers. |  |  |  |
| [96]  Ghana | To investigate the association with use of large-scale malaria  intervention | Cross-sectional survey | Children under-five | 2,449 |  | Not sleeping in IRS, ITN use did not have effect on malaria, mothers with lower education then secondary. | Malaria education through television is the best strategy to covey malaria education as it significantly reduces the odds of malaria infection. |  | RDT |
| [97]  Malawi | To compare the rate and factors associated with  ITN usage among children under 5 years of age, living in household with at least one ITN | Cross-sectional survey | Caregivers and children under-five years | 22,574 |  | Mothers with no or primary education, residing in female-headed households and residing in households that had poor supply of ITN are risk factors. | Increased ITN usage from 57.8% in 2010 to 69% in 2015. | No |  |
| [98]  Nigeria | To assess individual, household and community risk factors for malaria in Nigeria  To assess individual, household and community risk factors for malaria in Nigerian U5s. | Cross-sectional survey | Children under-five | 5,742 | 27% | Rural living, household size>7, the use of river and rainwater were risk factors. |  | No |  |
| [100]  Nigeria | To decompose changes in malaria prevalence amongst under-five children between 2003 and 2013.  To decompose changes in malaria prevalence amongst under-five children between 2003 and 2013 | Cross-sectional survey | Children under-five | 23,838 | 13.1% | ITN ownership and utilisation, |  |  |  |
| [101] | To examine the sociodemographic factors that determine malaria among under-five children in Ghana | Cross-sectional survey | Children under-five | 2,725 | 26% | Children <2 years, and No access to ITN. |  |  |  |
| [102]  Ghana | To examine the utilisation of ITN among caregivers of children under five years in Ho municipality of Ghana | Cross sectional survey | Caregivers of children under-five | 283 |  | Non-use of ITN is associate with perceived side effect of ITN and household number <5. |  |  |  |
| [103]  Ghana | To monitor the trend of ITN ownership and use and its impact on malaria and anaemia among children under five over the past decade in an area of intense, prolonged and seasonal malaria transmission | Cross-sectional surveys | Under five | 5,787 | 2006 – 40.4%  2010 - 33.2%  2015- 26.6% | Households who do not have nets at the beginning of raining season | Ownership of LLIN and its use together with other indicators of malaria prevalence generally im-proved five years after the implementation of a malaria control programme. |  |  |
| [104]  Ghana | To assess ownership, usage, effectiveness, knowledge, access and availability of ITNs among mothers with children under five in the Hohoe municipality. | Cross sectional survey | Children under five | 450 |  |  | Behaviour change communication strategies on ITN use lead to increase knowledge on availability and use of ITNs. |  |  |
| [105]  Burkina Faso | To estimate the level of domestic larval control practices (cleaning of the house and its surroundings, eradication of larval sources, and elimination of hollow objects that might collect water); | Cross-sectional survey | Children under-five | 1,705 | 26% |  | Domestic lavae control action and their integration into malaria prevention strategy, Ensuring universal access to ITN usage. Mothers’ participation in malaria information sessions increased the adoption of vector control actions and bed net use. Malaria prevalence was statistically lower among children in households where mothers had undertaken at least one vector control action or used bed-nets |  |  |
| [106]  Cameroon | To assess the effect of providing free malaria treatment in the Buea health district.  To assess the effect of providing free malaria treatment in the Buea health district. | Cross-sectional survey | Caregivers and children under-five |  |  |  | Increase utilisation of healthcare as general and malaria related consultations witnessed an increase after the implementation of free malaria treatment services. This was accounted for by improved and increased  testing, better reporting strategies which accompanied the free  malaria treatment package. |  |  |
| [116]  DR Congo | To examine knowledge, attitude, and practice on the use of ITNs in the prevention of malaria among pregnant women and guardians of children under five in the Democratic Republic of the Congo. | Cross sectional survey | Under five | 5,138 |  | Good attitude towards ITN usage |  |  |  |
| [117]  Cameroon | To determine the impact of the home-based management of childhood malaria morbidity and mortality in the Penka-Michel and Santchou Health Districts of the West Region in Cameroon | Cross-sectional survey | Children under five | 32,976 | 33.7% |  | The implementation of the integrated Community Directed Intervention (CDI) of malaria has benefited the population by enhancing early detection of childhood malaria cases.  Multi-skilled Community Health Workers were trained to strengthen the promotion and prevention of the disease through educational talks and home visits |  |  |
| [118]  Mali  Madagascar  Nigeria | To examined how malaria-related ideational factors may influence care-seeking behavior among female caregivers of children under five with fever | Cross-sectional survey | Children under-five care givers | 9,511 |  | Care givers who believed that it was their community norm to promptly seek care for children with malaria fever. Urban residents are likely to seek care and care givers with high level education are likely to seek care. |  |  |  |
| [119]  Dr congo**,** Uganda, Kenya | To estimate the true prevalence of malaria in children under five.  To estimate the true prevalence of malaria in children under five. | Cross Sectional survey | Children under five | 13,573 | 14.3% |  |  |  | Microscopy and RDTs |
| [84] 2011  Rwanda | To identify factors associated with Plasmodium infection and malaria in this population. | Cross sectional survey | Children under-five | 749 | PCR 16.7%  Microscopy11.7% | Children with age >3 years, children with fever, children with low weight, children with gastro-intestinal tract affection, children with respiratory tract infection, children with severe malnutrition |  |  | Microscopy  PCR |
| [99] et al, 2017  Zimbabwe | To determine factors associated with severe malaria so as to come up with evidence-based interventions to prevent severe malaria and associated mortality. | Case-control study | Children under five | 156 |  | Under nutrition, long distance (>10km) from homes to health facilities, and staying in a house under construction |  |  |  |
